# Real-world validation of the SLERPI diagnostic model with concordance and discordance analysis across established SLE classification criteria

**DOI:** 10.1186/s13075-026-03749-2

**Published:** 2026-02-10

**Authors:** Omima Ahmed El-Farra, Rasha Ali Abdel-Magied, Walaa Fawzy Mohamed, Mervat Eissa, Sarah Atef Sakr, Ghada A. Dawa, Amal Mohamed Elmesiry, Naglaa Shaban Elkholy, Mahmoud Risha, Doaa M. Sharaf, Muhammad Magdy Harb

**Affiliations:** 1 Rheumatology, Rehabilitation and Physical Medicine Department, Faculty of Medicine, Minia University, Minia, Egypt; 2 Rheumatology Department, Faculty of Medicine, Cairo University, Cairo, Egypt; 3 Rheumatology & Rehabilitation Department, Faculty of Medicine, Zagazig University, Zagazig, Egypt; 4 Rheumatology and Rehabilitation Department, Faculty of Medicine for Girls, Al-Azhar University, Cairo, Egypt; 5 Internal Medicine Department, Faculty of Medicine for Girls, Al-Azhar University, Cairo, Egypt; 6 Rheumatology & Rehabilitation Department, Faculty of Medicine, Al-Azhar University, Cairo, Egypt

**Keywords:** Systemic lupus erythematosus, SLERPI, Diagnostic accuracy, Classification criteria, Early disease, Concordance and discordance, Net reclassification improvement, Phenotypic heterogeneity

## Abstract

**Objective:**

Systemic lupus erythematosus (SLE) is a clinically heterogeneous disease in which early and atypical presentations frequently challenge existing classification frameworks. The Systemic Lupus Erythematosus Risk Probability Index (SLERPI) was developed as a probabilistic diagnostic aid, but its real-world performance relative to established classification criteria across disease phenotypes remains incompletely characterized.

**Methods:**

In this multicenter, cross-sectional study, we evaluated 1,281 participants, including 655 expert-confirmed SLE patients and 626 controls with other rheumatic diseases. Diagnostic performance of SLERPI, ACR-1997, SLICC-2012, and EULAR/ACR-2019 criteria was assessed against expert clinical diagnosis as the reference standard. Subgroup analyses were performed for early disease (≤ 1 year), sex, disease duration, and major organ involvement. Concordance and discordance between criteria were examined using UpSet plots, detailed phenotypic comparisons, and hierarchical cluster analysis of discordant cases. Net reclassification improvement (NRI) was used to quantify incremental diagnostic information.

**Results:**

All four systems demonstrated high diagnostic accuracy, with sensitivities ranging from 95.1–99.2% and specificities from 87.7–90.4%. SLERPI achieved the highest sensitivity (99.2%) and AUC (0.989), particularly excelling in early disease (≤ 1 year, sensitivity 98.0%, AUC 0.987). Net reclassification improvement favored SLERPI over ACR-1997 (+ 2.7%), SLICC-2012 (+ 1.6%), and EULAR/ACR-2019 (+ 4.3%). Concordance across systems was substantial, with 91.6% of patients classified by all four sets. Discordant cases (8.4%) revealed phenotype-specific patterns: ACR-1997 frequently missed immunologically active or hematologic-dominant cases, while EULAR/ACR-2019 underperformed in mucocutaneous-predominant disease. Cluster analysis identified four coherent subgroups, underscoring heterogeneity in missed classifications. SLERPI showed the lowest discordance, with residual misclassifications confined to hematologic-dominant phenotypes.

**Conclusion:**

SLE classification frameworks show substantial overlap in real-world practice, with discordance driven by phenotype-specific prioritization of disease domains rather than random failure. SLERPI complements established classification criteria by supporting identification of early and atypical SLE presentations, while traditional criteria remain essential for research standardization. Integrating probabilistic diagnostic tools with classification frameworks may enhance SLE recognition across diverse clinical contexts.

**Supplementary Information:**

The online version contains supplementary material available at 10.1186/s13075-026-03749-2.

## What is already known on this topic

- Systemic lupus erythematosus (SLE) is a heterogeneous autoimmune disease, making accurate classification challenging.
- Established classification criteria (ACR-1997, SLICC-2012, EULAR/ACR-2019) are widely used for research but have limitations in early or atypical disease presentations.
- The Systemic Lupus Erythematosus Risk Probability Index (SLERPI) was recently proposed as a weighted diagnostic model, but real-world validation across diverse patient populations has been limited.

## What does this study add?

- Provides large-scale, multicenter real-world validation of SLERPI against expert-confirmed SLE diagnoses.
- Highlights phenotype-specific patterns of discordance across classification systems, showing that missed cases are not random but reflect differential weighting of clinical and immunological domains.
- Identifies coherent clusters of discordant patients, underscoring the biological heterogeneity of SLE and the complementary strengths of different diagnostic frameworks.

## How might this study affect research, practice or policy?

- Supports SLERPI as a valuable adjunct to established criteria, especially for early recognition and atypical disease presentations in clinical practice.
- Reinforces the continued role of traditional classification systems for research standardization, while suggesting integration of SLERPI to improve case capture.
- Provides evidence for phenotype-specific limitations in current frameworks, informing refinement of future diagnostic and classification tools.
- Encourages longitudinal and multi-ethnic validation studies to guide policy on harmonizing diagnostic models across clinical and research settings.

## Introduction

Systemic lupus erythematosus (SLE) is a chronic autoimmune disease with multisystem involvement and diverse autoantibody profiles. The diagnosis of SLE remains complex, relying largely on expert judgment. Although many patients are identified by typical manifestations and serology, a proportion present with incomplete or evolving features that delay diagnosis [1]. This diagnostic uncertainty has underscored the need for standardized classification criteria to facilitate uniform patient selection for clinical trials [2]. The 1997 American College of Rheumatology (ACR) criteria [3], while highly specific are now recognized for their suboptimal sensitivity, particularly in early and mild disease presentations [4]. This limitation prompted the development of the 2012 Systemic Lupus International Collaborating Clinics (SLICC) criteria [5], which broadened the clinical and immunologic spectrum, and the more recent 2019 European Alliance of Associations for Rheumatology (EULAR)/ACR criteria [6], which introduced a hierarchical, weighted approach aimed at earlier classification.

Despite these advances, challenges remain. Each criterion set reflects a distinct conceptual approach. ACR-1997 prioritizes specificity, SLICC-2012 seeks balance between clinical and immunologic domains, and EULAR/ACR-2019 applies a weighted, score-based approach [7]. Consequently, real-world studies show that a meaningful proportion of patients, especially those with early disease, incomplete phenotypes, or predominant single-organ involvement, remain unclassified or variably classified across systems [8].

The Systemic Lupus Erythematosus Risk Probability Index (SLERPI) was developed to address these gaps as a probabilistic, weighted scoring tool designed to reflect the continuum of SLE risk [9]. Initial studies suggest that SLERPI performs favorably compared to conventional criteria [10–13]. However, its added value relative to each of the three established classification criteria has not been comprehensively evaluated.

The aim of this study was to validate the SLERPI tool in a real-world, multicenter cohort and to evaluate its diagnostic performance alongside established classification frameworks, with a focus on early disease recognition, concordance, discordance, and phenotype-specific patterns.

## Patients and methods

### Study design and setting

A multicenter, cross-sectional diagnostic accuracy study was performed between October 2024 and August 2025 at rheumatology outpatient clinics at the tertiary hospitals affiliated with Cairo University, Minia University, Zagazig University, and Al-Azhar University (Al-Hussein and Al-Zahraa Hospitals), in Egypt. The study followed the STARD guidelines, complying with the Declaration of Helsinki. Informed consent was obtained from all participants. The protocol was approved by the Institutional Research Board of Faculty of Medicine, Minia University (approval number:1328–10–2024).

### Patient recruitment and study population

A total of 1,517 potentially eligible participants were systematically screened across five academic medical centers, comprising 753 suspected SLE cases and 764 potential disease controls. The complete participant flow, including screening numbers, exclusions with specific reasons, and final enrollment, is detailed in Supplementary Fig. 1.

Both SLE cases and controls underwent diagnostic evaluation using a standardized protocol across all participating centers. The reference standard consisted of diagnostic consensus between two expert rheumatologists, each with more than 10 years of clinical experience in autoimmune rheumatic diseases. The experts were not responsible for the initial enrolment or routine clinical care of the patients included in the study, thereby reducing the risk of selection and confirmation bias.

Each expert independently reviewed the complete clinical dataset, including detailed medical history, physical examination findings, laboratory investigations, and imaging results, as documented in the patients’ medical records. Experts were blinded to each other’s assessment.

Each expert provided a binary diagnostic determination (SLE present vs. absent) based on their overall clinical judgment. Diagnostic consensus was predefined as a complete agreement between the two experts. Disagreements were resolved by joint review. Patients diagnosed with other autoimmune rheumatic diseases served as control group.

Participants were systematically excluded based on the following predetermined criteria:

(1) inadequate clinical or serological documentation precluding comprehensive evaluation, (2) pregnancy or lactation status, (3) current or previous malignancy, and (4) failure to achieve diagnostic consensus between the two expert rheumatologists. The following variables were documented: age at enrollment, sex, age at symptom onset, disease duration, all features relevant to established classification criteria, including: malar rash, discoid rash, photosensitivity, oral or nasal ulcerations, non-erosive arthritis, serositis, renal involvement (defined as proteinuria > 0.5g/24h or presence of cellular casts), neurological manifestations, hematologic abnormalities (leukopenia, lymphopenia, thrombocytopenia, or hemolytic anemia), fever, and alopecia.

Serological evaluation encompassed: Antinuclear Antibodies (ANA) were determined using Indirect Immunofluorescence on HEp-2 cells, with the endpoint titer being recorded, anti-dsDNA was considered positive if it met one of the following two conditions: A positive result by the high-specificity *Crithidia luciliae* Immunofluorescence Test, or a positive result from a quantitative solid-phase immunoassay ELISA that was 2 times the laboratory's Upper Limit of Normal, anti-Smith antibodies (anti-Sm), antiphospholipid antibodies (including lupus anticoagulant, anti-cardiolipin IgM/IgG, and anti-β2-glycoprotein-I IgM/IgG), complement levels (C3 and C4), and direct antiglobulin test (Coomb’s test).

For subgroup analysis, early SLE was defined as a disease duration of ≤ 1 year from the onset of the first symptom attributable to SLE. Patients with a disease duration of > 1 year were classified as having established SLE [14].

Each participant was independently evaluated against the following four classification criteria sets:

1. *ACR-1997*: Classification required fulfillment of ≥ 4 out of 11 criteria [3].
2. *SLICC-2012*: Classification required fulfillment of ≥ 4 criteria, with at least one clinical and one immunological criterion, or biopsy-proven lupus nephritis in the presence of ANA or anti-dsDNA antibodies [5].
3. *EULAR/ACR-2019*: Classification required an ANA titer of ≥ 1:80 as an obligatory entry criterion, followed by an additive weighted score; a total score of ≥ 10 was used to classify a patient as having SLE [6].
4. *SLERPI*: Classification was based on a cut-off score of ≥ 7 points from its weighted index of clinical and immunological features [9]. Each item was scored when present and when not better explained by an alternative diagnosis (e.g., infection, drug reaction). The final SLERPI score ranged from 0–14 points, with scores ≥ 7 classified as meeting SLERPI criteria for SLE, and scores < 7 classified as not meeting criteria. The SLERPI criteria definitions and weight are summarized in Supplementary Table 1.

### Statistical analysis

Statistical analyses were performed using IBM SPSS Statistics version 28 and R Studio version 4.3.2. Continuous variables were expressed as mean (standard deviation) and categorical variables as frequencies and percentages. Group comparisons were made using Student’s t-test and Chi-square test. Diagnostic performance metrics including sensitivity, specificity, positive predictive value, negative predictive value, and area under the receiver operating characteristic curve (AUC) with 95% confidence intervals were calculated. Net reclassification improvement (NRI) analysis quantified the incremental diagnostic value of SLERPI. Hierarchical cluster analysis using Euclidean distance and Ward’s method with using dendrogram to show process of clustering. All statistical tests were two-sided, with *p <* 0.05 considered statistically significant.

## Results

### Patient characteristics

A total of 1,281 participants were included, comprising 655 patients with expert-confirmed SLE and 626 disease controls diagnosed with other rheumatic diseases (Supplementary Table 2). Patients with SLE were younger at enrolment than controls (32.8 ± 9.7 vs. 42.4 ± 11.7 years), had earlier symptom onset (25.7 ± 8.5 vs. 33.8 ± 10.8 years), and a shorter disease duration (7.2 ± 6.3 vs. 8.5 ± 7.1 years; all *p <* 0.001). The SLE cohort was predominantly female (93.4% vs. 78.3% in controls, *p <* 0.001). Controls mainly had rheumatoid arthritis (53.5%), systemic sclerosis (17.7%), and Behçet’s disease (11.8%), and smaller proportions of other connective tissue diseases.

All clinical and immunological features comprising the SLERPI index were significantly more prevalent in the SLE group versus controls (*p <* 0.001 for all features). The most discriminative features included proteinuria (60.9% vs. 1.9%), malar or maculopapular rash (74.5% vs. 4.8%), and alopecia (66.4% vs. 11.0%). Immunologically, ANA positivity was nearly universal among SLE patients (99.2% vs. 41.7% in controls, *p <* 0.001). Neurological involvement was documented in 139 SLE patients (21.2%), including seizures (12.1%), delirium (3.4%), and other manifestations such as psychosis, myelitis, or peripheral neuropathy (5.8%).

The SLE cohort was subdivided into 99 patients with early disease (≤ 1 year from symptom onset) and 556 had established disease. While the prevalence of most features was consistent between these subgroups, patients with early SLE exhibited a numerically higher prevalence of alopecia (74.7% vs. 64.9%) and cytopenias, while consumed complement (42.4% vs. 60.1%) and chronic cutaneous lupus (4.0% vs. 12.8%) were less frequent.

### Performance characteristics of SLERPI and classification criteria

Performance metrics for SLERPI and the three classification criteria were evaluated using expert clinical diagnosis as the reference standard (Table 1).

**Table 1:** Diagnostic performance in early (SLE *N =* 99) vs overall (SLE *N =* 655) cohort

Classification Criterion	Sensitivity % (95% CI)	Specificity % (95% CI)	PPV % (95% CI)	NPV % (95% CI)	Accuracy % (95% CI)	AUC (95% CI)
***Early Disease Cohort (Disease Duration* ≤ *1 year)***						
ACR-1997	91.92 (84.70–96.45)	90.42 (87.83–92.61)	60.26 (51.99–68.13)	98.61 (97.27–99.40)	90.62 (88.26–92.64)	0.967 (0.953–0.980)
SLICC-2012	96.97 (91.40–99.37)	88.34 (85.56–90.75)	56.80 (48.98–64.39)	99.46 (98.43–99.89)	89.52 (87.06–91.65)	0.975 (0.963–0.986)
EULAR/ACR-2019	96.97 (91.40–99.37)	87.70 (84.87–90.17)	55.49 (47.76–63.03)	99.46 (98.42–99.89)	88.97 (86.46–91.15)	0.970 (0.957–0.984)
SLERPI	97.98 (92.89–99.75)	88.98 (86.26–91.32)	58.43 (50.54–66.02)	99.64 (98.71–99.96)	90.21 (87.81–92.27)	0.987 (0.980–0.993)
***Overall Disease Cohort***						
ACR-1997	95.11 (93.17–96.63)	90.42 (87.83–92.61)	91.22 (88.84–93.23)	94.65 (92.53–96.31)	92.82 (91.26–94.17)	0.974 (0.967–0.981)
SLICC-2012	98.32 (97.02–99.16)	88.34 (85.56–90.75)	89.82 (87.37–91.93)	98.05 (96.54–99.02)	93.44 (91.95–94.74)	0.981 (0.975–0.986)
EULAR/ACR-2019	96.18 (94.42–97.52)	87.70 (84.87–90.17)	89.11 (86.58–91.31)	95.64 (93.64–97.16)	92.04 (90.42–93.46)	0.977 (0.970–0.983)
SLERPI	99.24 (98.23–99.75)	88.98 (86.26–91.32)	90.40 (88.01–92.46)	99.11 (97.94–99.71)	94.22 (92.80–95.44)	0.989 (0.985–0.993)

Early disease cohort includes SLE patients with disease duration ≤ 12 months (*n =* 99) and all controls (*n =* 626), Overall cohort includes all SLE patients and controls

95% CIs calculated using exact binomial method (sensitivity, specificity, PPV, NPV) and DeLong method (AUC). Expert clinical diagnosis served as the reference standard

*PPV* Positive Predictive Value, *NPV* Negative Predictive Value, *AUC* Area Under the Curve

In the overall cohort, all four systems demonstrated high sensitivity (95.1–99.2%) and specificity (87.7–90.4%). SLERPI showed the highest sensitivity (99.24%, 95% CI 98.23–99.75) and AUC (0.989, 95% CI 0.985–0.993), while the classification criteria showed slightly lower but overlapping performance ranges.

In the early SLE subgroup (≤ 12 months, *n =* 99), sensitivities ranged from 91.9% (ACR-1997) to 98.0% (SLERPI), with all systems maintaining high negative predictive values (> 98%). Discriminative ability remained excellent across tools (AUC 0.967–0.987).

### Performance by disease duration

Sensitivity trends across disease duration categories are shown in Supplementary Fig. 2. In early disease patients (*n =* 99), sensitivities ranged from 91.9% to 98% and in patients with disease duration of 1–5 years (*n =* 276), sensitivities increased across all systems (94.2–99.6%). In long-standing disease (> 5 years), all systems demonstrated high sensitivity (97.1–100%). An unexpected decline in sensitivity of the EULAR/ACR-2019 criteria was observed in patients with disease duration > 10 years, potentially reflecting incomplete documentation of transient early manifestations such as fever or arthralgia.

### Sex-stratified and organ-based analyses

Performance patterns were similar in female and male patients (Supplementary Table 3). In males, SLICC-2012, EULAR/ACR-2019, and SLERPI classified all SLE cases, while ACR-1997 classified 90.7%. Specificity and accuracy were comparable across sexes.

Across major organ involvement subgroups (neurological, renal, hematological), all systems demonstrated consistently high sensitivity (Supplementary Table 4). Newer criteria and SLERPI maintained high sensitivity across diverse organ-dominant phenotypes, with specificity remaining stable.

### ROC curve analysis

ROC analysis confirmed excellent discriminative ability for all systems (Supplementary Fig. 3). SLERPI demonstrated the highest AUC in both the overall cohort (0.989) and early SLE subgroup (0.987), with closely overlapping curves for the classification criteria.

### Net reclassification improvement

NRI analysis evaluated changes in case classification when applying SLERPI relative to each classification system (Table 2). The total NRI was 2.7% versus ACR-1997 (30 SLE cases correctly reclassified upward, 3 downward), 1.6% versus SLICC-2012 (7 cases up, 1 down), and 4.3% versus EULAR/ACR-2019 (24 cases up, 4 down).

**Table 2:** Net Reclassification Improvement (NRI)

Comparison	NRI_Events	NRI_NonEvents	NRI_Total	Cases_Up	Cases_Down	Controls_Up	Controls_Down
SLERPI vs ACR-1997	0.041 (4.1%)	−0.014 (−1.4%)	**0.027 (2.7%)**	30 (4.6%)	3 (0.5%)	20 (3.2%)	11 (1.8%)
SLERPI vs SLICC-2012	0.009 (0.9%)	0.006 (0.6%)	**0.016 (1.6%)**	7 (1.1%)	1 (0.2%)	11 (1.8%)	15 (2.4%)
SLERPI vs EULAR/ACR-2019	0.031 (3.1%)	0.013 (1.3%)	**0.043 (4.3%)**	24 (3.7%)	4 (0.6%)	17 (2.7%)	25 (4.0%)

NRI Events = Net reclassification improvement in SLE cases

NRI Non-Events = Net reclassification improvement in controls

Total NRI = NRI Events + NRI Non-Events

Positive values indicate improvement with SLERPI

### Concordance and discordance between criteria

**Fig. 1:**
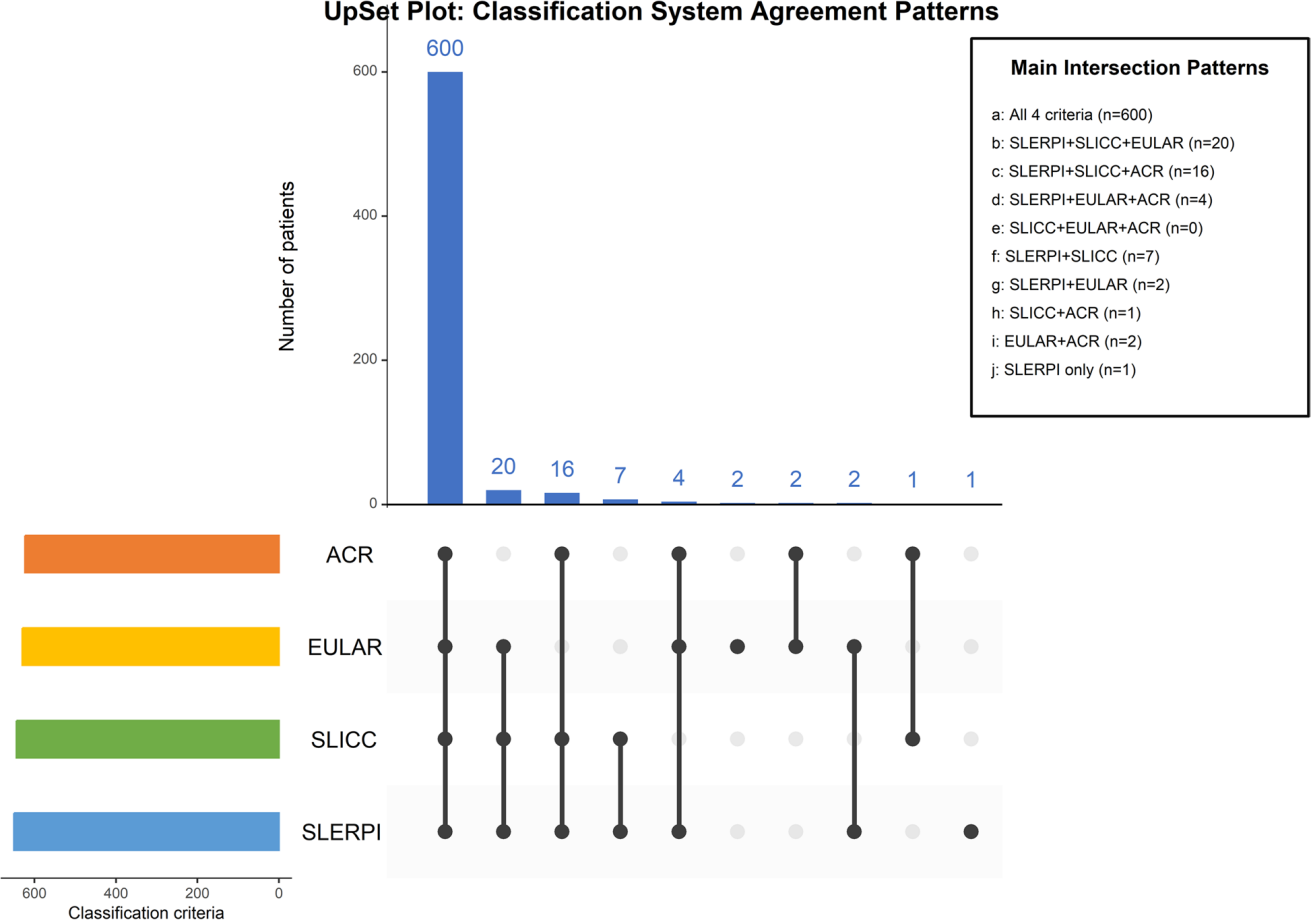
This UpSet plot displays the specific combinations of classification systems that identified SLE patients. The bar height shows the number of patients for each combination pattern, with dots and connecting lines below indicating which systems were involved

Overall concordance among the four systems was high. As shown in the UpSet plot (Fig. 1), 600 patients (91.6%) were classified by all four systems. The largest discordant subset comprised patients classified by SLERPI, SLICC-2012, and EULAR/ACR-2019 but not ACR-1997 (*n =* 20). Only three patients were classified by a single system alone (SLERPI *n =* 1; EULAR/ACR-2019 *n =* 2).

### Characteristics of discordant cases

**Fig. 2:**
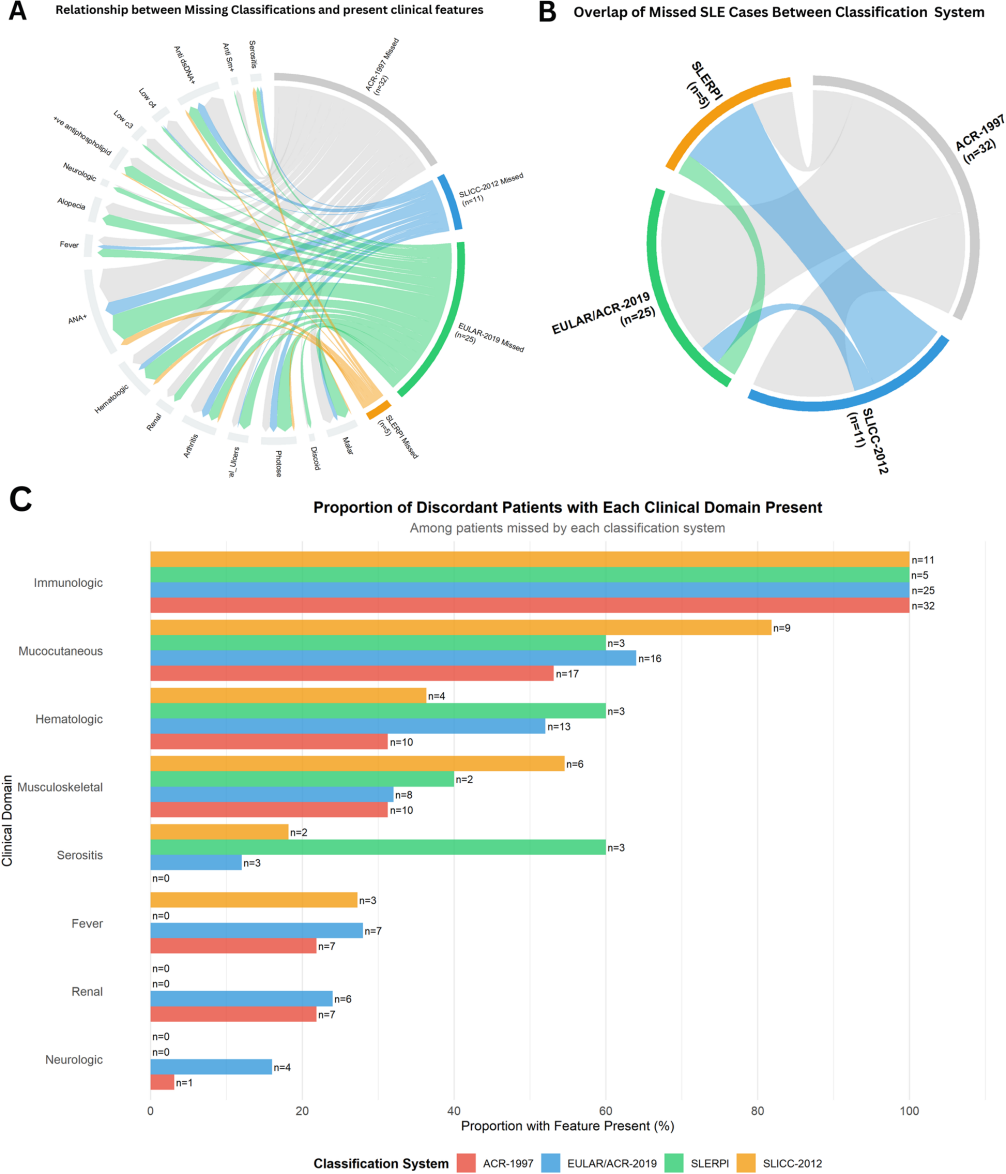
Patterns of discordance across SLE classification systems: **A**. Chord diagram illustrating the clinical and serological features present among SLE patients missed by each classification system. Outer arcs represent the four systems, scaled by the number of missed patients: ACR-1997 (*n =* 32), EULAR/ACR-2019 (*n =* 25), SLICC-2012 (*n =* 11), and SLERPI (*n =* 5). Ribbons connect each system to the features observed in its missed cases, with ribbon width proportional to feature frequency. **B** Chord diagram depicting overlap among patients missed by different classification systems. Ribbon width reflects the number of shared missed cases between systems. Substantial overlap is observed between ACR-1997 and EULAR/ACR-2019, while fewer shared missed cases involve SLICC-2012. All patients missed by SLERPI were also missed by at least one other system. **C** Stacked bar chart showing the proportion of clinical and serological domains present among patients missed by each classification system, highlighting phenotype-specific patterns of discordance

Among the 655 expert-confirmed SLE patients, 55 individuals (8.4%) were discordantly classified by at least one system. Only 27% of these patients were missed by two or more criteria (Fig. 2, Supplementary Table 5).

Patients missed by ACR-1997 frequently exhibited immunological activity (anti-dsDNA positivity and complement consumption) and mucocutaneous manifestations but lacked high-weight organ involvement such as serositis or neuropsychiatric disease. EULAR/ACR-2019 most commonly failed to classify patients with mucocutaneous-predominant presentations, including photosensitivity, malar rash, and oral ulcers, reflecting the lower weighting of these features within its scoring framework. SLICC-2012 missed a smaller subset of patients, typically those with milder or incomplete multisystem involvement. The 5 patients missed by SLERPI had scores narrowly below the diagnostic threshold (median score 6.5). None of these patients were uniquely missed by SLERPI alone, underscoring substantial overlap with discordance observed in other classification criteria.

### Cluster analysis of discordant patients

**Fig. 3:**
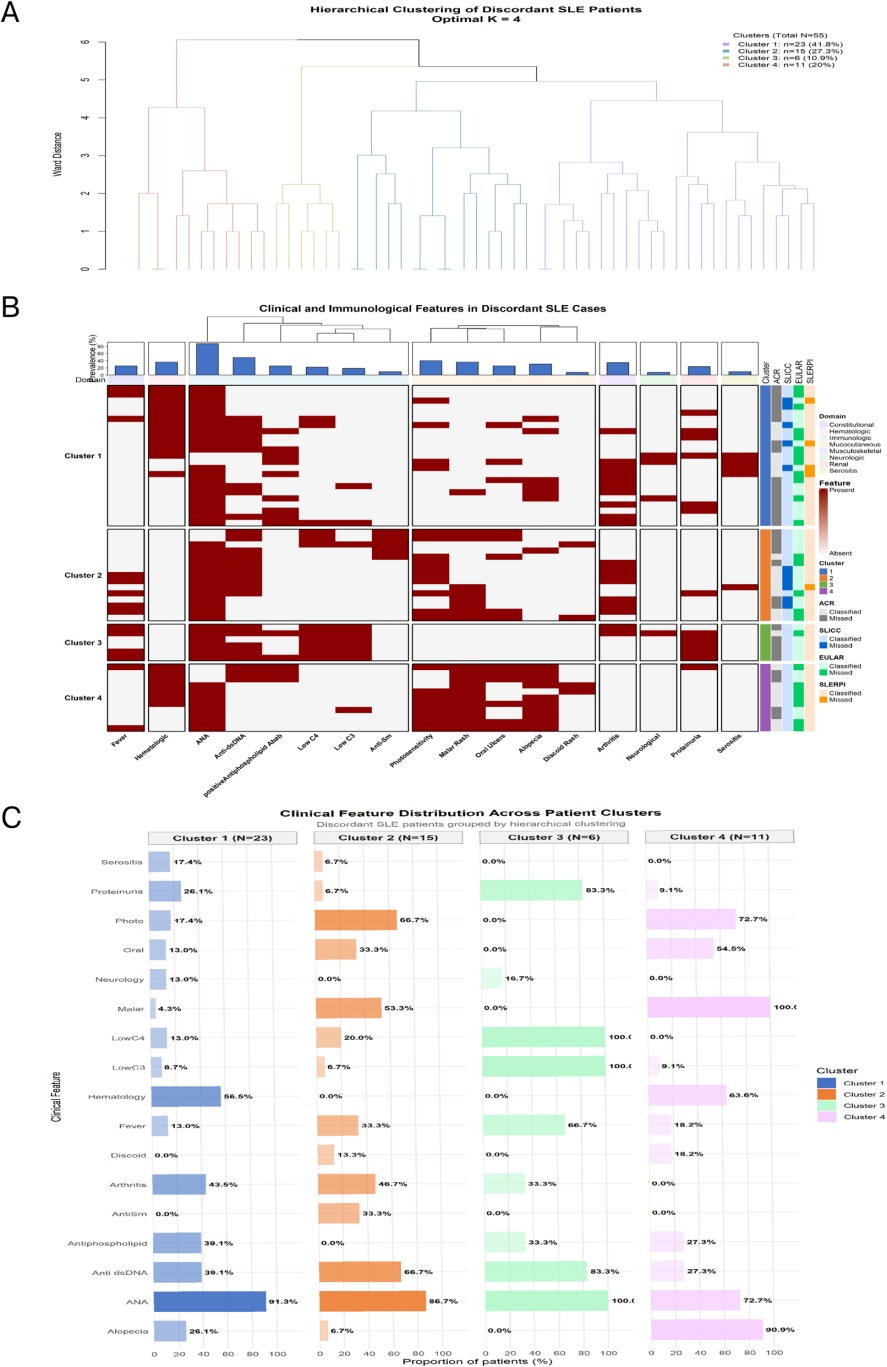
Phenotypic clustering of discordant SLE cases (*n =* 55). **A** Hierarchical clustering dendrogram of discordant SLE patients based on clinical and serological features using Ward’s linkage. Four phenotypically coherent clusters were identified, with hematologic-dominant disease forming the most distinct subgroup. **B** Heatmap of clinical and immunological features across discordant patients, organized by cluster. Feature presence is shown in red and absence in white; annotation bars indicate classification outcomes across criteria sets. **C** Distribution of key clinical and serological features across clusters. Cluster 1 shows predominance of hematologic and antiphospholipid features, Cluster 2 a classic mucocutaneous–serologic phenotype, Cluster 3 isolated immunological activity with minimal clinical manifestations, and Cluster 4 mucocutaneous-limited disease. ANA positivity was high across all clusters

Cluster analysis of 55 discordant SLE cases identified four biologically coherent phenotypic subgroups (Fig. 3, Supplementary Table 6). The largest subgroup, Cluster 1 (*n =* 23, 41.8%), was characterized by hematologic-dominant disease with frequent antiphospholipid antibody positivity and relatively limited mucocutaneous involvement. Cluster 2 (*n =* 15, 27.3%) represented a classic mucocutaneous–serologic phenotype, combining universal mucocutaneous manifestations with high anti-dsDNA positivity. Cluster 3 (*n =* 6, 10.9%) exhibited isolated immunological activity, marked by complement consumption, anti-dsDNA positivity, and proteinuria, but minimal clinical organ involvement, consistent with an immunologically active yet clinically pauci-symptomatic profile. Cluster 4 (*n =* 11, 20.0%) showed a mucocutaneous-limited phenotype with prominent cutaneous features and little extracutaneous disease.

The distribution of missed classifications differed markedly across clusters. ACR-1997 showed the highest discordance overall, particularly in immunologically active or hematologic-dominant phenotypes (Clusters 3 and 1), whereas EULAR/ACR-2019 more frequently failed to classify mucocutaneous-limited and hematologic-predominant cases (Clusters 4 and 1). SLICC-2012 demonstrated lower overall discordance but missed a subset of patients with prominent mucocutaneous and serologic activity (Cluster 2). In contrast, SLERPI showed the lowest miss rates across all clusters, with residual unclassified cases largely confined to Cluster 1 (Supplementary Table 6).

## Discussion

The demographic characteristics of our cohort align well with established epidemiological patterns of SLE. The female predominance observed in our SLE cohort (93.4%) is consistent with the typical 9:1 female-to-male ratio reported in large population-based studies and registry data [15]. The prevalence of major organ involvement in our cohort also mirrors published literature, with renal involvement affecting 60.9% of patients, which falls within the reported range of 50–70% across diverse ethnic populations [16, 17]. Similarly, the frequency of hematologic involvement (50.8%) are comparable to rates described in international cohorts and systematic reviews [11, 18]. The neuropsychiatric involvement is similar to other Egyptian cohorts [19, 20]. However, the frequency of neuropsychiatric involvement vary according to the recruitment center (primary versus tertiary) and in different ethnic populations [21], this heterogeneity may affect the performance of different SLE criteria sets in different cohorts.

In this large multicenter cohort of 1,281 participants, we demonstrated that SLERPI exhibited the highest overall diagnostic performance among all evaluated criteria sets. SLERPI achieved near-perfect sensitivity (99.24%) and high specificity (89.0%). SLICC-2012 and EULAR/ACR-2019 also showed excellent sensitivity (98.3% and 96.2%, respectively), while ACR-1997 demonstrated the highest specificity but lower sensitivity. These findings are consistent with prior validation studies and meta-analyses, confirming the progressive improvement in sensitivity achieved by newer classification systems [8].

Balancing sensitivity and specificity remains a central challenge in SLE classification due to the disease’s clinical heterogeneity and fluctuating course. In research settings, maximizing sensitivity to ensure inclusion of true SLE cases is often prioritized over avoiding false positives [22]. The EULAR/ACR and SLICC classification criteria prioritize high sensitivity to include early SLE cases without incurring a substantial loss in specificity compared to ACR-1997 [23]. SLERPI further optimizes this trade-off, achieving superior sensitivity with only a modest reduction in specificity, resulting in the highest overall accuracy among the evaluated tools. This performance profile supports SLERPI’s suitability for both clinical and research settings [24].

Our results are in line with the meta-analyses by Lu et al. [8] and Lerkvaleekul et al. [25] as well as the original SLICC [5] and EULAR [6] validation studies. The SLERPI sensitivity in the Chinese cohort [10] matched our sensitivity estimates, and the SLERPI validation reported an accuracy of 94.8%, which closely matches the accuracy seen in our cohort [9]. A large Egyptian data-based validation study has shown that SLERPI had excellent diagnostic efficacy and specificity. Supplementary Table 7 demonstrates a comparison of SLE classification criteria performance across different cohorts.

Early SLE diagnosis remains particularly challenging, as patients often present with incomplete clinical phenotypes and evolving serologic abnormalities. Consistent with prior meta-analyses by Lu et al. [8] and Lerkvaleekul et al. [25] meta-analyses, we observed that contemporary criteria outperform ACR-1997 in early disease. Notably, SLERPI demonstrated the highest sensitivity (98.0%) and the highest positive predictive value in patients with disease duration ≤ 1 year, confirming its strength in early disease classification. These findings closely align with prior early-SLE evaluations [9, 23].

The superior performance of SLERPI in early disease likely reflects its probabilistic, weighted scoring system, which captures subtle clinical and laboratory abnormalities that may not independently satisfy categorical thresholds in traditional criteria. Because early SLE is characterized by diagnostic uncertainty and incomplete manifestations, a mathematical model that integrates partial information may better reflect real-world clinical reasoning [9].

The phenotypic differences in SLE presentation between the sexes are probably reflected in criteria performance, as male patients are more likely to present with classical, severe manifestations that easily meet conventional classification criteria which is supported by the meta-analysis by Boodhoo [26] which showed that renal manifestation and serositis were higher in males than female and supported by CSTAR registry and Chan et al. study [27, 28].

Among male participants (*n =* 179; 43 SLE, 136 controls), SLICC-2012, EULAR/ACR-2019, and SLERPI each identified all SLE cases, yielding a sensitivity of 100%, whereas ACR-1997 showed slightly lower sensitivity. Specificity was high and identical across all criteria in males (94.9–95.6%), resulting in comparable overall accuracy. The uniformly high sensitivity observed among males for SLICC-2012, EULAR/ACR-2019, and SLERPI should be interpreted cautiously given the smaller male sample size.

To our knowledge, this is the first application of net reclassification improvement (NRI) to SLE classification and diagnostic tools, demonstrating that SLERPI may capture additional expert-confirmed SLE cases who fall below traditional classification thresholds, while showing largely comparable case capture to SLICC-2012.

A key strength of this study lies in its detailed concordance and discordance analyses. The high concordance observed among the four classification systems provides strong validation of the underlying disease construct. More than 90% of expert-confirmed SLE patients were classified concordantly by all criteria, indicating that despite methodological differences, these systems converge on a shared core SLE phenotype. This reinforces the validity of expert diagnosis as a reference standard and supports the biological coherence of SLE across classification frameworks.

Importantly, discordant cases were not random or preclinical. Instead, they represented established, biologically meaningful phenotypes that clustered into coherent subgroups. ACR-1997 most frequently missed patients with immunologically active and mucocutaneous-dominant disease lacking major organ involvement, reflecting its limited weighting of serologic activity and non-scarring cutaneous features. EULAR/ACR-2019 preferentially missed patients with mucocutaneous and musculoskeletal manifestations that failed to reach weighted scoring thresholds. SLICC-2012 missed fewer cases overall but still failed to capture some patients with limited multi-domain involvement.

These clusters indicates that discordance arises from how individual systems weight and prioritize specific disease domains, rather than from random misclassification. Importantly, patients not classified by SLERPI generally had scores just below the diagnostic threshold. Ease of use is a critical determinant of real-world applicability. SLERPI was specifically designed as a simple, clinician-friendly scoring tool, applying 14 weighted clinical and serologic items with a single threshold (score > 7) for SLE classification. This design preserves interpretability while maintaining high sensitivity, making it more practical for daily use than the domain-restricted, weighted EULAR/ACR-2019 or the lengthy SLICC-2012 framework. SLERPI’s usability is comparable to the historic simplicity of the ACR-1997 4-of-11 rule while delivering markedly better diagnostic performance [9].

It is essential to emphasize that ACR-1997, SLICC-2012, and EULAR/ACR-2019 were developed as classification instruments intended to standardize patient selection for clinical trials, where maximizing specificity and minimizing heterogeneity are paramount. Their performance characteristics should therefore be interpreted within that context. In contrast, SLERPI was designed as a diagnostic aid to support clinical decision-making, particularly in early or evolving disease. Our findings underscore that differences observed between these tools largely reflect differences in intended purpose rather than inherent methodological superiority. SLERPI may be better suited for real-world diagnostic evaluation, whereas the older criteria remain valuable for ensuring homogeneity in clinical trial cohorts.

This multicenter, real-world study includes a large, expert-confirmed SLE cohort spanning early and established disease, enhancing clinical relevance and diagnostic validity. The use of complementary analytical approaches including subgroup analyses, net reclassification improvement, and detailed concordance–discordance evaluation with phenotypic clustering provides insight not only into overall performance but also into where and why different tools diverge. These features strengthen interpretability, transparency, and applicability of the findings to routine clinical practice.

Several limitations merit consideration. Its cross-sectional design precludes longitudinal assessment of disease evolution and limits evaluation of how classification performance changes over time. Recruitment from tertiary care centers in Egypt may restrict generalizability to other healthcare settings. In addition, documentation of transient early manifestations such as fever and mucocutaneous symptoms was less complete in patients with long-standing disease, which may have slightly affected EULAR/ACR-2019 sensitivity. Finally, the control group consisted exclusively of non-SLE rheumatologic diseases, whereas real-world diagnostic challenges often include infectious and malignant mimics.

In conclusion, this study provides a comprehensive real-world evaluation of SLE diagnostic and classification frameworks, emphasizing concordance, discordance, and phenotypic heterogeneity. Our findings support the complementary roles of SLERPI and established classification criteria, with SLERPI offering value for early and atypical disease recognition, and traditional criteria continuing to serve as robust tools for research standardization. Future longitudinal and multi-ethnic studies are warranted to further define how these tools can be optimally integrated across clinical and research settings.

## Supplementary Information

Supplementary Material 1: Supplementary Figure 1. Patient recruitment flowchart. Supplementary Figure 2. Performance by Disease Duration (Documentation Quality Analysis): The line plot illustrates sensitivity trends across disease duration categories. Supplementary Figure 3. ROC curve analysis of the 4 criteria sets in overall cohort (panel A) and early cohort (panel B).

Supplementary Material 2: Supplementary Table 1. SLERPI criteria weighted scores. Supplementary table 2. Characteristics of the study cohort (*n=*1281). Supplementary Table 3. Diagnostic Performance Stratified by Sex. Supplementary Table 4. Diagnostic Performance by Organ System Involvement. Supplementary Table 5. Clinical Features Present Among Patients Missed by Each Classification System. Supplementary Table 6. Clinical and serological characteristics of discordant SLE patient. Supplementary table 7. Comparison of SLE classification criteria performance across different cohorts.

## Data Availability

The datasets supporting the conclusions of this article are available from the corresponding author on reasonable request.
