# Supplementary material for: Real-world validation of the SLERPI diagnostic model with concordance and discordance analysis across established SLE classification criteria
Source: Arthritis Res Ther. 2026 Feb 10;28:60. doi: 10.1186/s13075-026-03749-2 (PMC12930877; doi:10.1186/s13075-026-03749-2)
Supplement: Supplementary file 1 — Supplementary Material 1: Supplementary Figure 1. Patient recruitment flowchart. Supplementary Figure 2. Performance by Disease Duration (Documentation Quality Analysis): The line plot illustrates sensitivity trends across disease duration categories. Supplementary Figure 3. ROC curve analysis of the 4 criteria sets in overall cohort (panel A) and early cohort (panel B). [file 13075_2026_3749_MOESM1_ESM.zip › SupplFig1_Flow Chart.pptx]

## Slide 1
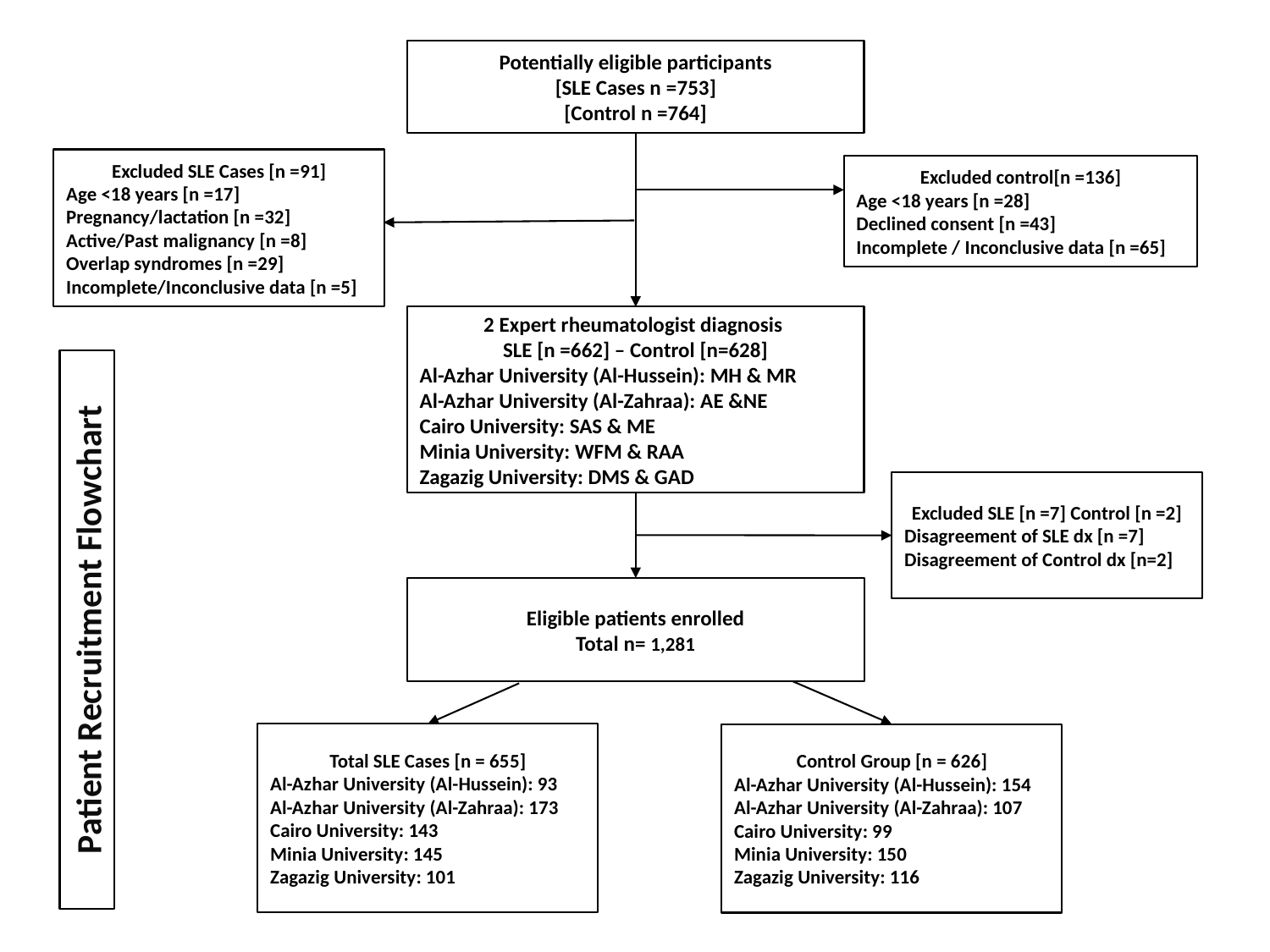

Potentially eligible participants
[SLE Cases n =753]
[Control n =764]
Excluded SLE Cases [n =91]
Age <18 years [n =17]
Pregnancy/lactation [n =32]
Active/Past malignancy [n =8]
Overlap syndromes [n =29]
Incomplete/Inconclusive data [n =5]
Excluded control[n =136]
Age <18 years [n =28]
Declined consent [n =43]
Incomplete / Inconclusive data [n =65]
2 Expert rheumatologist diagnosis
SLE [n =662] – Control [n=628]
Al-Azhar University (Al-Hussein): MH & MR
Al-Azhar University (Al-Zahraa): AE &NE
Cairo University: SAS & ME
Minia University: WFM & RAA
Zagazig University: DMS & GAD
Excluded SLE [n =7] Control [n =2]
Disagreement of SLE dx [n =7]
Disagreement of Control dx [n=2]
Eligible patients enrolled
Total n= 1,281
Patient Recruitment Flowchart
Total SLE Cases [n = 655]
Al-Azhar University (Al-Hussein): 93
Al-Azhar University (Al-Zahraa): 173
Cairo University: 143
Minia University: 145
Zagazig University: 101
Control Group [n = 626]
Al-Azhar University (Al-Hussein): 154
Al-Azhar University (Al-Zahraa): 107
Cairo University: 99
Minia University: 150
Zagazig University: 116
