# Supplementary figures and images for: Real-world validation of the SLERPI diagnostic model with concordance and discordance analysis across established SLE classification criteria
Source: Arthritis Res Ther. 2026 Feb 10;28:60. doi: 10.1186/s13075-026-03749-2 (PMC12930877; doi:10.1186/s13075-026-03749-2)

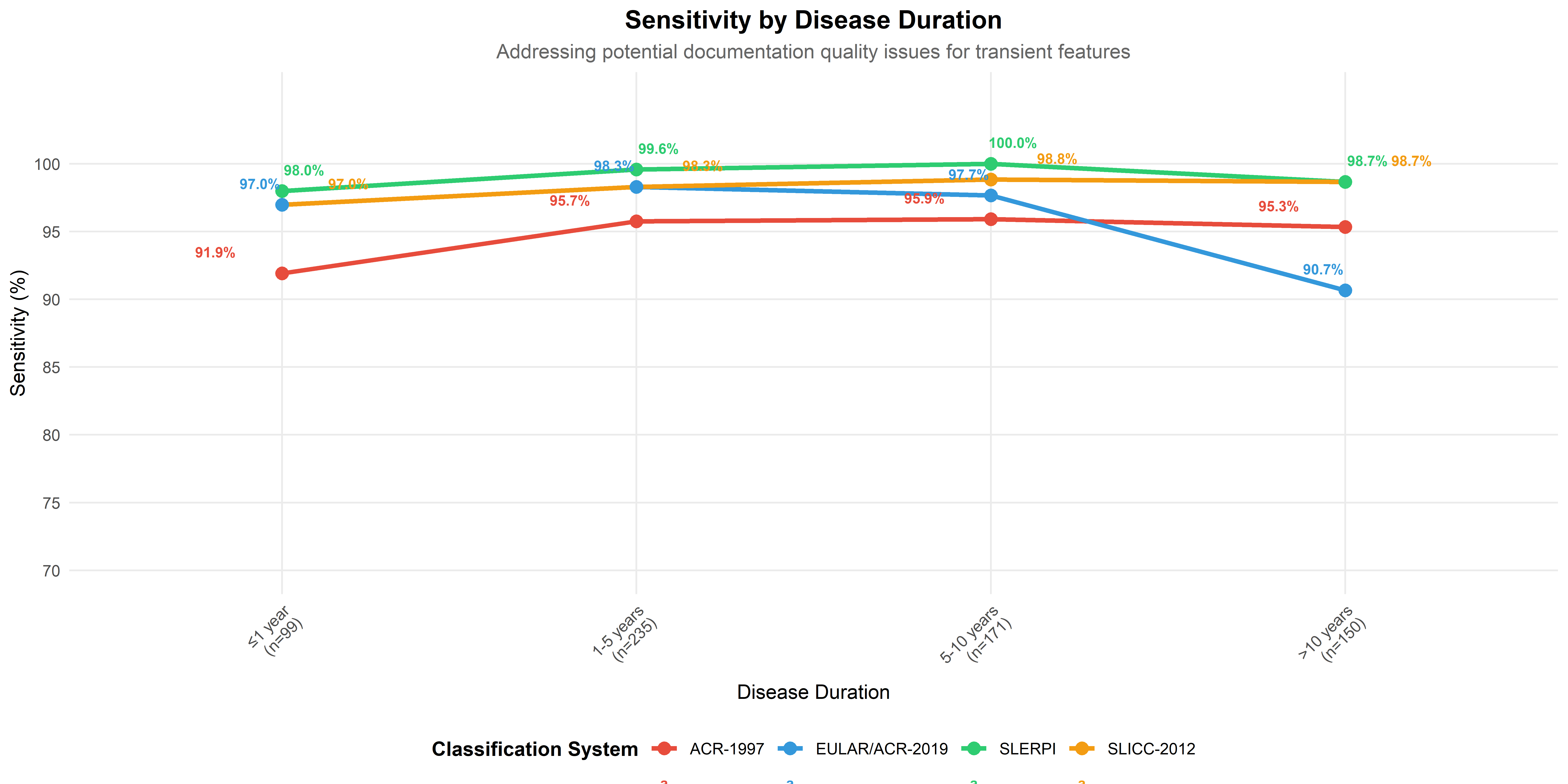

Supplement: Supplementary file 1 — Supplementary Material 1: Supplementary Figure 1. Patient recruitment flowchart. Supplementary Figure 2. Performance by Disease Duration (Documentation Quality Analysis): The line plot illustrates sensitivity trends across disease duration categories. Supplementary Figure 3. ROC curve analysis of the 4 criteria sets in overall cohort (panel A) and early cohort (panel B). [file 13075_2026_3749_MOESM1_ESM.zip › SupplFigure2_DurationSensitivity.png]

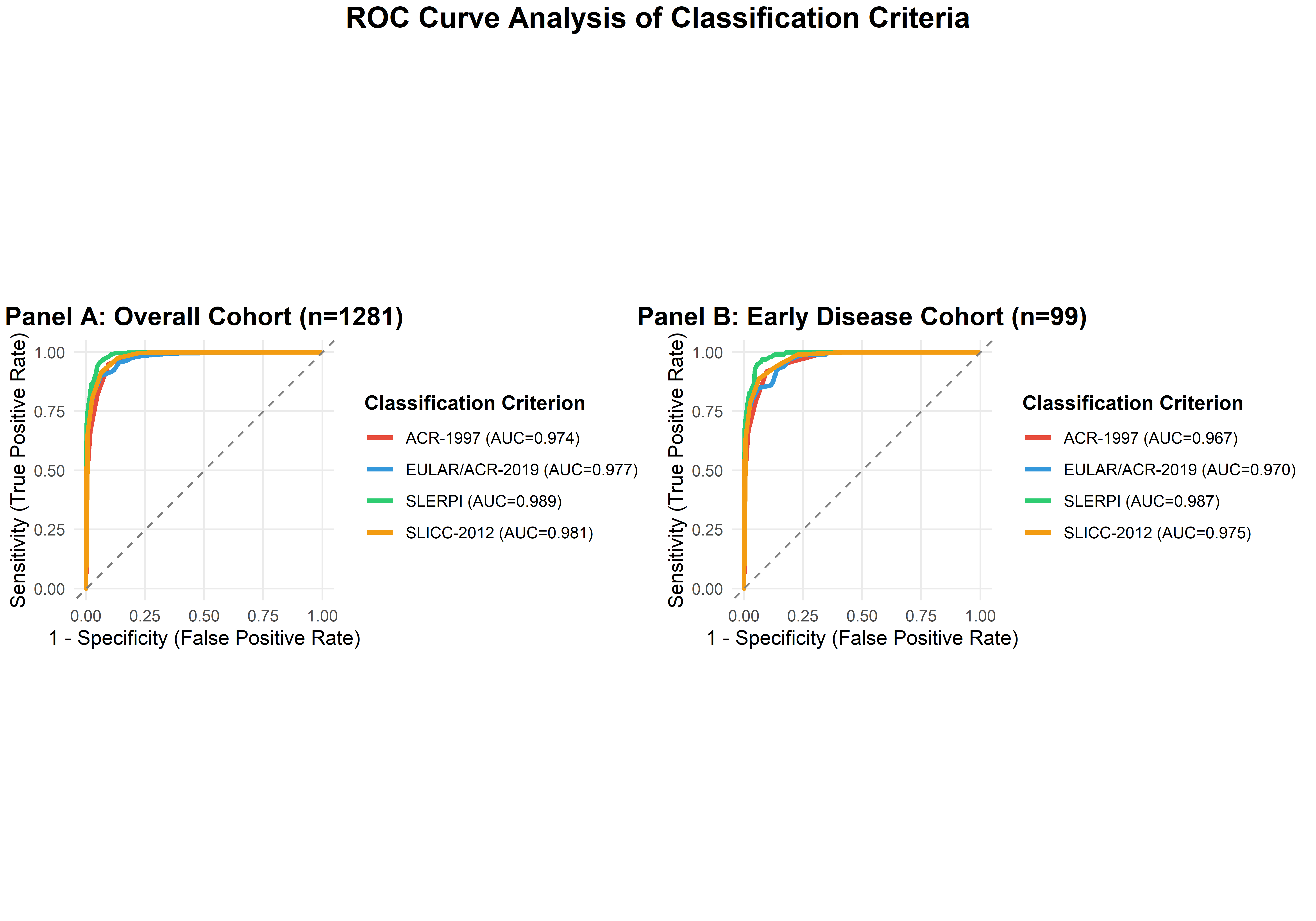

Supplement: Supplementary file 1 — Supplementary Material 1: Supplementary Figure 1. Patient recruitment flowchart. Supplementary Figure 2. Performance by Disease Duration (Documentation Quality Analysis): The line plot illustrates sensitivity trends across disease duration categories. Supplementary Figure 3. ROC curve analysis of the 4 criteria sets in overall cohort (panel A) and early cohort (panel B). [file 13075_2026_3749_MOESM1_ESM.zip › SupplFigure3_ROC.png]
