# Supplementary material for: Real-world validation of the SLERPI diagnostic model with concordance and discordance analysis across established SLE classification criteria
Source: Arthritis Res Ther. 2026 Feb 10;28:60. doi: 10.1186/s13075-026-03749-2 (PMC12930877; doi:10.1186/s13075-026-03749-2)
Supplement: Supplementary file 2 — Supplementary Material 2: Supplementary Table 1. SLERPI criteria weighted scores. Supplementary table 2. Characteristics of the study cohort (n=1281). Supplementary Table 3. Diagnostic Performance Stratified by Sex. Supplementary Table 4. Diagnostic Performance by Organ System Involvement. Supplementary Table 5. Clinical Features Present Among Patients Missed by Each Classification System. Supplementary Table 6. Clinical and serological characteristics of discordant SLE patient. Supplementary table 7. Comparison of SLE classification criteria performance across different cohorts. [file 13075_2026_3749_MOESM2_ESM.docx]

Supplementary Table 1 – SLERPI criteria weighted scores

|  | Clinical/Immunological Feature | Definition and Diagnostic Criteria | Weighted Score |
| --- | --- | --- | --- |
| 1 | Malar or Maculopapular Rash | Documented erythema of the cheeks or diffuse maculopapular eruption | 3.0 |
| 2 | Subacute Cutaneous Lupus or Discoid Lupus (SCLE/DLE) | Characteristic annular/papulosquamous or discoid lesions | 2.0 |
| 3 | Alopecia | Non-scarring hair loss temporally associated with disease activity | 1.5 |
| 4 | Mucosal Ulcers | Painless oral or nasal mucosal ulceration | 1.0 |
| 5 | Arthritis (Synovitis) | Clinical non-erosive synovitis | 2.0 |
| 6 | Serositis | Pleuritis or pericarditis documented clinically or radiologically | 1.5 |
| 7 | Leucopenia | White blood cell count ≤4,000/µL on at least one occasion | 1.5 |
| 8 | Thrombocytopenia or Autoimmune Hemolytic Anemia (AIHA) | Platelet count ≤100×10⁹/L or laboratory evidence of immune hemolysis | 4.5 |
| 9 | Neurologic Disorder | Seizures, psychosis, transverse myelitis, or other neuropsychiatric manifestations attributable to SLE per standard definitions | 1.5 |
| 10 | Proteinuria | Protein excretion ≥0.5 g/24 h or equivalent spot urine protein/creatinine ratio | 4.5 |
| 11 | Antinuclear Antibody (ANA) Positivity | By immunofluorescence ≥1:80 or validated positive ELISA | 3.0 |
| 12 | Low Complement (C3 and/or C4) | Serum C3 and/or C4 below the laboratory reference range | 2.0 |
| 13 | Immunological Disorder | Presence of anti-dsDNA, anti-Sm, or antiphospholipid antibodies (including anti-β2-glycoprotein where reported) | 2.5 |
| 14 | Interstitial Lung Disease (ILD) | Radiologic evidence on high-resolution CT | –1.0 |

Supplementary table 2: Characteristics of the study cohort (n=1281)

| Characteristic | Controls (n=626) | SLE Cases (n=655) | | | | p-value |
| --- | --- | --- | --- | --- | --- | --- |
|  |  | Total (n=655) | Early SLE ≤1 year (n=99) | | Established SLE >1 year (n=556) |  |
| **Demographics** | | | |  |  |  |
| Age at enrolment, years | 42.44 (11.68) | 32.84 (9.68) | 26.62 (9.30) | | 32.84 (9.68) | **<0.001** |
| Age at symptom onset, years | 33.78 (10.80) | 25.74 (8.47) | 25.65 (9.57) | | 25.74 (8.47) | **<0.001** |
| Disease duration, years | 8.54 (7.11) | 7.19 (6.26) | 0.8 (0.30) | | 7.19 (6.26) | **<0.001** |
| Female sex | 490 (78.3%) | 612(93.4%) | 91 (91.9%) | | 521 (93.7%) | **<0.001** |
| **SLERPI Features** | | | |  |  |  |
| Malar/maculopapular rash | 30 (4.8%) | 488 (74.5%) | 73 (73.3%) | | 415 (74.6%) | **<0.001** |
| SCLE/DLE | 2 (0.3%) | 75 (11.5%) | 4 (4%) | | 71 (12.8%) | **<0.001** |
| Alopecia | 69 (11%) | 435 (66.4%) | 74 (74.7%) | | 361 (64.9%) | **<0.001** |
| Mucosal ulcers | 145 (23.2%) | 416 (63.5%) | 68 (68.7%) | | 348 (62.6%) | **<0.001** |
| Arthritis | 449 (71.7%) | 415  (63.4%) | 65 (65.7%) | | 350 (62.9%) | **0.001** |
| Serositis | 75 (12%) | 235(35.9%) | 37 (37.4%) | | 198 (35.6%) | **<0.001** |
| Neurological disorder | 19 (3%) | 139 (21.2%) | 17 (17.2%) | | 122 (21.9%) | **<0.001** |
| Leucopenia | 29 (4.6%) | 289 (44.1%) | 52 (52.5%) | | 237 (42.6%) | **<0.001** |
| Thrombocytopenia/AIHA | 20 (3.2%) | 224 (34.2%) | 42 (42.4%) | | 182 (32.7%) | **<0.001** |
| Proteinuria | 12 (1.9%) | 399 (60.9%) | 51 (51.5%) | | 348 (62.6%) | **<0.001** |
| ANA positivity | 261 (41.7%) | 650 (99.2%) | 99 (100%) | | 551 (99.1%) | **<0.001** |
| Low complement (C3 and C4) | 10 (1.6%) | 376 (57.4%) | 42 (42.4%) | | 334 (60.1%) | **<0.001** |
| Immunological disorder | 16 (2.6%) | 548 (83.7%) | 84 (84.9%) | | 464 (83.5%) | **<0.001** |
| Interstitial lung disease | 136 (21.7%) | 43 (6.6%) | 6 (6.1%) | | 37 (6.7%) | **<0.001** |

Values are presented as mean (SD) or n (%). p-values compare total SLE versus controls using Student’s t-test for continuous variables and chi-square test for categorical variables. Bold p values are statistically significant at p<0.05. AIHA, autoimmune hemolytic anemia; ANA, antinuclear antibody; DLE, discoid lupus erythematosus; SCLE, subacute cutaneous lupus erythematosus; SLE, systemic lupus erythematosus; SLERPI, systemic lupus erythematosus risk probability index. Early SLE was defined as disease duration ≤1 year from symptom onset; established SLE as >1 year. Control group comprised 335 (53.5%) rheumatoid arthritis, 111 (17.7%) systemic sclerosis,74 (11.8%) Behçet disease**,** 37 (5.9%) Sjögren disease, 36 (5.8%) dermatomyositis, 13 (2.1%) mixed connective tissue disease, 11 (1.8%) autoimmune thyroiditis and 9 (1.4%) autoimmune hepatitis.

*Supplementary Table 3. Diagnostic Performance Stratified by Sex*

| Sex | Classification Criterion | SLE Cases | Controls | Sensitivity % (95% CI) | Specificity % (95% CI) | PPV % (95% CI) | NPV % (95% CI) | Accuracy % (95% CI) |
| --- | --- | --- | --- | --- | --- | --- | --- | --- |
| Male | ACR-1997 | 43 | 136 | 90.70 (77.86-97.41) | 95.59 (90.64-98.36) | 86.67 (73.21-94.95) | 97.01 (92.53-99.18) | 94.41 (89.97-97.29) |
|  | SLICC-2012 |  |  | 100.00 (91.78-100.00) | 94.85 (89.68-97.91) | 86.00 (73.26-94.18) | 100.00 (97.18-100.00) | 96.09 (92.11-98.41) |
|  | EULAR/ACR-2019 |  |  | 100.00 (91.78-100.00) | 94.85 (89.68-97.91) | 86.00 (73.26-94.18) | 100.00 (97.18-100.00) | 96.09 (92.11-98.41) |
|  | **SLERPI** |  |  | **100.00 (91.78-100.00)** | **94.85 (89.68-97.91)** | **86.00 (73.26-94.18)** | **100.00 (97.18-100.00)** | **96.09 (92.11-98.41)** |
| Female | ACR-1997 | 612 | 490 | 95.42 (93.46-96.94) | 88.98 (85.87-91.61) | 91.54 (89.10-93.58) | 93.97 (91.40-95.95) | 92.56 (90.85-94.04) |
|  | SLICC-2012 |  |  | 98.20 (96.81-99.10) | 86.53 (83.18-89.43) | 90.10 (87.58-92.26) | 97.47 (95.52-98.73) | 93.01 (91.34-94.45) |
|  | EULAR/ACR-2019 |  |  | 95.92 (94.03-97.34) | 85.71 (82.30-88.69) | 89.35 (86.73-91.60) | 94.38 (91.82-96.33) | 91.38 (89.56-92.97) |
|  | **SLERPI** |  |  | **99.18 (98.10-99.73)** | **87.35 (84.07-90.16)** | **90.73 (88.28-92.82)** | **98.85 (97.33-99.62)** | **93.92 (92.34-95.26)** |
| *PPV = Positive Predictive Value; NPV = Negative Predictive Value* | | | | | | | | |
| *95% CIs calculated using exact binomial method (Clopper-Pearson)* | | | | | | | | |
| *Expert clinical diagnosis served as the reference standard* | | | | | | | | |

*Supplementary Table 4. Diagnostic Performance by Organ System Involvement*

| Organ System | Classification Criterion | SLE Cases with Involvement | Sensitivity % (95% CI) | Specificity % (95% CI) | PPV % (95% CI) | NPV % (95% CI) | Accuracy % (95% CI) |
| --- | --- | --- | --- | --- | --- | --- | --- |
| Neurological | ACR-1997 | 130 | 99.23 (95.79-99.98) | 90.42 (87.83-92.61) | 68.25 (61.10-74.82) | 99.82 (99.02-100.00) | 91.93 (89.76-93.77) |
|  | SLICC-2012 |  | 100.00 (97.20-100.00) | 88.34 (85.56-90.75) | 64.04 (57.02-70.64) | 100.00 (99.34-100.00) | 90.34 (88.01-92.35) |
|  | EULAR/ACR-2019 |  | 96.92 (92.31-99.16) | 87.70 (84.87-90.17) | 62.07 (55.01-68.77) | 99.28 (98.16-99.80) | 89.29 (86.86-91.40) |
|  | **SLERPI** |  | **100.00 (97.20-100.00)** | **88.98 (86.26-91.32)** | **65.33 (58.27-71.92)** | **100.00 (99.34-100.00)** | **90.87 (88.59-92.83)** |
| Renal | ACR-1997 | 396 | 98.23 (96.39-99.29) | 90.42 (87.83-92.61) | 86.64 (83.14-89.65) | 98.78 (97.50-99.51) | 93.44 (91.75-94.88) |
|  | SLICC-2012 |  | 100.00 (99.07-100.00) | 88.34 (85.56-90.75) | 84.43 (80.83-87.60) | 100.00 (99.34-100.00) | 92.86 (91.10-94.36) |
|  | EULAR/ACR-2019 |  | 98.48 (96.73-99.44) | 87.70 (84.87-90.17) | 83.51 (79.83-86.76) | 98.92 (97.66-99.60) | 91.88 (90.03-93.48) |
|  | **SLERPI** |  | **100.00 (99.07-100.00)** | **88.98 (86.26-91.32)** | **85.16 (81.60-88.27)** | **100.00 (99.34-100.00)** | **93.25 (91.53-94.71)** |
| Hematological | ACR-1997 | 333 | 97.00 (94.55-98.55) | 90.42 (87.83-92.61) | 84.33 (80.30-87.83) | 98.26 (96.83-99.16) | 92.70 (90.87-94.27) |
|  | SLICC-2012 |  | 98.80 (96.95-99.67) | 88.34 (85.56-90.75) | 81.84 (77.72-85.49) | 99.28 (98.17-99.80) | 91.97 (90.07-93.61) |
|  | EULAR/ACR-2019 |  | 96.10 (93.42-97.91) | 87.70 (84.87-90.17) | 80.60 (76.37-84.38) | 97.69 (96.08-98.76) | 90.62 (88.59-92.39) |
|  | **SLERPI** |  | **99.10 (97.39-99.81)** | **88.98 (86.26-91.32)** | **82.71 (78.63-86.29)** | **99.46 (98.44-99.89)** | **92.49 (90.64-94.08)** |
| *Controls (all subgroups): n=626*  *PPV = Positive Predictive Value; NPV = Negative Predictive Value*  *95% CIs calculated using exact binomial method (Clopper-Pearson)*  *Each subgroup includes SLE patients with specific organ involvement plus all controls* | | | | | | | |
| *Expert clinical diagnosis served as the reference standard* | | | | | | | |

*Supplementary Table 5. Clinical Features Present Among Patients Missed by Each Classification System*

| Clinical Domain | Specific Feature | ACR-1997 (N=32 missed) | EULAR/ACR-2019 (N=25 missed) | SLERPI (N=5 missed) | SLICC-2012 (N=11 missed) |
| --- | --- | --- | --- | --- | --- |
| **Hematologic** | Hematologic | 10 (31.2%) | 13 (52.0%) | 3 (60.0%) | 4 (36.4%) |
| **Immunologic** | ANA positive | 28 (87.5%) | 22 (88.0%) | 5 (100.0%) | 11 (100.0%) |
|  | Anti dsDNA | 16 (50.0%) | 8 (32.0%) | 2 (40.0%) | 6 (54.5%) |
|  | Low C4 | 10 (31.2%) | 2 (8.0%) | 0 (0.0%) | 1 (9.1%) |
|  | Low C3 | 9 (28.1%) | 2 (8.0%) | 0 (0.0%) | 0 (0.0%) |
|  | +ve Antiphospholipid Ab | 8 (25.0%) | 9 (36.0%) | 1 (20.0%) | 0 (0.0%) |
|  | Anti Sm | 4 (12.5%) | 1 (4.0%) | 0 (0.0%) | 0 (0.0%) |
| **Mucocutaneous** | Alopecia | 10 (31.2%) | 11 (44.0%) | 0 (0.0%) | 0 (0.0%) |
|  | Malar rash | 9 (28.1%) | 10 (40.0%) | 1 (20.0%) | 3 (27.3%) |
|  | Photosensitivity | 6 (18.8%) | 12 (48.0%) | 2 (40.0%) | 6 (54.5%) |
|  | Oral ulcers | 5 (15.6%) | 9 (36.0%) | 0 (0.0%) | 1 (9.1%) |
|  | Discoid rash | 1 (3.1%) | 3 (12.0%) | 0 (0.0%) | 0 (0.0%) |
| **Musculoskeletal** | Arthritis | 10 (31.2%) | 8 (32.0%) | 2 (40.0%) | 6 (54.5%) |
| **Neurologic** | Neurological | 1 (3.1%) | 4 (16.0%) | 0 (0.0%) | 0 (0.0%) |
| **Renal** | Proteinuria | 7 (21.9%) | 6 (24.0%) | 0 (0.0%) | 0 (0.0%) |
| **Serositis** | Serositis | 0 (0.0%) | 3 (12.0%) | 3 (60.0%) | 2 (18.2%) |
| Values are presented as number (percentage) of patients missed by each classification system in whom the specified feature was present. | | | | | |

Supplementary Table 6. Clinical and serological characteristics of discordant SLE patient clusters

| Characteristic | Cluster 1 | Cluster 2 | Cluster 3 | Cluster 4 |
| --- | --- | --- | --- | --- |
| **Cluster Description** | **Hematologic/Antiphospholipid-dominant** | **Classic Mucocutaneous/Serologic-dominant** | **immunological abnormalities** | **Mucocutaneous- limited** |
| N | 23 | 15 | 6 | 11 |
| Age (years), mean ± SD | 35.8 ± 9.8 | 36.2 ± 9.3 | 32.5 ± 10.8 | 33.2 ± 8.3 |
| Female, n (%) | 23 (100.0%) | 13 (86.7%) | 4 (66.7%) | 11 (100.0%) |
| Disease Duration (years), mean ± SD | 10.2 ± 10.9 | 7.3 ± 6.7 | 8.3 ± 7.8 | 7.1 ± 5.1 |
| Early Disease, n (%) | 6 (26.1%) | 1 (6.7%) | 1 (16.7%) | 2 (18.2%) |
| Mucocutanous, n (%) | 10 (43.5%) | 15 (100.0%) | 0 (0.0%) | 11 (100.0%) |
| Arthritis, n (%) | 10 (43.5%) | 7 (46.7%) | 2 (33.3%) | 0 (0.0%) |
| Neurology, n (%) | 3 (13.0%) | 0 (0.0%) | 1 (16.7%) | 0 (0.0%) |
| Serositis, n (%) | 4 (17.4%) | 1 (6.7%) | 0 (0.0%) | 0 (0.0%) |
| Fever, n (%) | 3 (13.0%) | 5 (33.3%) | 4 (66.7%) | 2 (18.2%) |
| Antiphospholipid antibodies, n (%) | 9 (39.1%) | 0 (0.0%) | 2 (33.3%) | 3 (27.3%) |
| Low C3, n (%) | 2 (8.7%) | 1 (6.7%) | 6 (100.0%) | 1 (9.1%) |
| Low C4, n (%) | 3 (13.0%) | 3 (20.0%) | 6 (100.0%) | 0 (0.0%) |
| Anti-Sm+, n (%) | 0 (0.0%) | 5 (33.3%) | 0 (0.0%) | 0 (0.0%) |
| Proteinuria, n (%) | 6 (26.1%) | 1 (6.7%) | 5 (83.3%) | 1 (9.1%) |
| Hematological, n (%) | 13 (56.5%) | 0 (0.0%) | 0 (0.0%) | 7 (63.6%) |
| ANA+, n (%) | 21 (91.3%) | 13 (86.7%) | 6 (100.0%) | 8 (72.7%) |
| Anti-dsDNA+, n (%) | 9 (39.1%) | 10 (66.7%) | 5 (83.3%) | 3 (27.3%) |
| Missed by ACR-1997, n (%) | 16 (69.6%) | 7 (46.7%) | 5 (83.3%) | 4 (36.4%) |
| Missed by SLICC-2012, n (%) | 5 (21.7%) | 6 (40.0%) | 0 (0.0%) | 0 (0.0%) |
| Missed by EULAR/ACR-2019, n (%) | 12 (52.2%) | 5 (33.3%) | 1 (16.7%) | 7 (63.6%) |
| Missed by SLERPI, n (%) | 4 (17.4%) | 1 (6.7%) | 0 (0.0%) | 0 (0.0%) |
| Data are presented as mean ± standard deviation or number (%). “Early disease” was defined as disease duration ≤1 year at diagnosis. Mucocutaneous involvement includes malar rash, photosensitivity, oral ulcers, alopecia, and other cutaneous manifestations. Hematologic involvement includes leukopenia, lymphopenia, thrombocytopenia, or hemolytic anemia. Missed by criteria indicates failure to meet the respective classification or diagnostic threshold despite expert-confirmed SLE. Expert clinical diagnosis served as the reference standard. | | | | |

Supplementary table 7: comparison of SLE classification criteria performance across different cohorts

| Accuracy (%) | 84.9 (NA) | 89.0 (NA) | 91.8 (NA) | 94.8 (92.8-96.4) | 93.3 (91.0, 95.5) | | 94.4 (91.7, 97.1) | 91.4 (88.3, 94.5) | 91.5 (88.3, 94.8) | 89.6 (87.1, 91.7)  95.0 | 95.0 (93.1, 96.4) 94.3 | 94.3 (92.4, 95.9) | 93.6 (91.9, 95.4)  ACR, | 94.9 (93.2 – 96.3) | 95.8 (94.3 – 97.1) | 92.8 (90.9 – 94.5) | 94.1 (92.3 – 95.6) | 92.82 (91.26-94.17) | 93.4 (91.9–94.7) | 92.0 (90.4–93.5) | 94.22 (92.80-95.44) |
| --- | --- | --- | --- | --- | --- | --- | --- | --- | --- | --- | --- | --- | --- | --- | --- | --- | --- | --- | --- | --- | --- |
| Specificity (%) | 93.7 (NA) | 86.0 (NA) | 95.2 (NA) | 93.7 (88.0-96.9) | 95.9 (90.8, 98.7) | | 90.2 (83.6, 94.9) | 87.8 (80.7, 93.0) | 84.6 (76.9, 90.4) | 96.4 (94.0, 98.0) | 92.2 (89.1, 95.7) | 91.4 (88.2, 94.0) | 89.4 (85.8, 92.2) | 98.8 (97.3 – 99.6) | 97.2 (95.2 – 98.5) | 93.3 (90.5 – 95.4) | 92.8 (89.9 – 95.0) | 90.42 (87.83-92.61) | 88.3 (85.6–90.6) | 87.7 (84.9–90.0) | 88.98 (86.26-91.32) |
| Sensitivity (%) | 82.4 (NA) | 90.4 (NA) | 90.8 (NA) | 95.1 (92.8-96.8) | 90.6 (87.3, 93.3) | | 98.5 (96.7, 99.4) | 94.9 (92.3, 96.9) | 98.5 (96.7, 99.4) | 82.1 (77.7, 86.0) | 98.0 (95.9, 99.2) | 97.4 (95.2, 98.8) | 98.3 (96.3, 99.4) | 91.0 (87.9 – 93.5) | 94.5 (91.9 – 96.4) | 92.4 (89.5 – 94.7) | 95.4 (93.0 – 97.2) | 95.11 (93.17-96.63) | 98.3 (97.0–99.0) | 96.2(94.4–97.4) | 99.24 (98.23-99.75) |
| Criterion | ACR-1997 | SLICC-2012 | EULAR/ACR-2019 | SLERPI ≥7 | ACR-1997 | | SLICC-2012 | EULAR/ACR-2019 | SLERPI ≥7 | ACR-1997 | SLICC-2012 | EULAR/ACR-2019 | SLERPI ≥7 | ACR-1997 | SLICC-2012 | EULAR/ACR-2019 | SLERPI ≥7 | ACR-1997 | SLICC-2012 | EULAR/ACR-2019 | SLERPI ≥7 |
| Key Inclusion Features & Collection Method | Data from Rheumatology Clinics (Heraklion and Attikon University | | | | | Retrospective audit from single tertiary hospital. SLE from (ALRB) | | | | Electronic medical records at 2^nd^ Affiliated Hospital of Soochow University | | | | Data randomly selected from a database at (CREA) in Bogota, Colombia. | | | | Real-World Multicentric study from rheumatology clinics at 3ry University hospitals across Egypt. | | | |
| F% | 91.0 % SLE/ 88.8% controls | | | | | 84.6% female (Overall) | | | | 91.5% SLE/ 72.5% controls | | | | 91.3% SLE/ 85.1%  controls | | | | 93.4% SLE/ 78.3% Controls | | | |
| Sample Size  (N SLE / N Control) | 512 SLE / 143 Controls (N=655) (Validation Cohort) | | | | | 394 SLE / 123 Controls (N=517) | | | | 352 SLE / 385 Controls (N=737) | | | | 435 SLE / 430 Controls (N=865) | | | | 655 SLE / 626 Controls (N=1281) | | | |
| Cohort | European Cohort (SLERPI Derivation/Validation)^9^ | | | | | Australian Cohort ^12^ | | | | Chinese  Cohort ^10^ | | | | Colombian Cohort^11^ | | | | Egyptian Cohort  (this work) | | | |
